# High-Land Malarial or Mushroom Fever

**Published:** 1879-11

**Authors:** J. G. Westmoreland

**Affiliations:** Atlanta, Ga.


### HIGH-LAND MALARIAL OR MUSHROOM FEVER.

By J. G. Westmoreland, M.D., Atlanta, Ga.

During the past summer and up to this time, a fever of peculiar type and in some nrighborhoods of malignant character, has prevailed in this and several surronnding counties. In some portions of Paulding, Polk, Coob and Cherokee Counties the mortality has amounted to fifty per cent. of the cases. It is remarkable that quite a number of cases have occurred in Fulton county, but very few, if any, within the city limits of Atlanta. It seems, therefore, that in this instance, at any rate, the densely populated city has proved more healthy than the surrounding sparsely settled country, where no cesspools nor stinking sewers are known. Strange, doubtless, this will appear to those finding fever in every sewer and diphtheria in every gutter around town.

To those accustomed to marsh malarial fever the disease referred to comes with surprise. On the highest, dry, and ordinarily salubrious sections, this fever prevails with severity, equal to that of low, damp localities. The great questions with the people and with doctors are, what is it, and from what cause does it come? It does not assume the usual characteristics of typhoid fever in every respect, nor is it controlled by quinine so perfectly as intermittent or remittent fever. While the nervous disturbance somewhat resembles that of typhoid, yet the usual duration, being only two weeks, makes it differ essentially from this disease.

In 1847 the same fever, exactly, prevailed in some counties of Middle Georgia, and again in 1866, the identical peculiarities were observed in a form of fever which prevailed to some extent in the neighborhood of Atlanta.

During the months of July and August, 1847, and in the same months of the present year, the greatest number of mushrooms were seen all over the uncultivated land of the forest. So abundant were they in both of the years referred to that casual observers wondered at the immense number of "frog-stools" seen in the woods. The experience of 1847 gave the writer good reason for predicting troublesome fever to follow the appearance of the large crop of cryptogams in 1879, and it has been signally verified.

It is, threfore, reasonable to suppose the fever in question has had its cause in the germs or sporules of the numerous cryptogams mentioned. It is known that the mushroom is the growth of a night and has the duration of a day. .Falling down, the innumerable spores rise and are wafted by the breeze so as to infect the atmosphere in the neighborhood around. The fever coming on at a suitable time after the breaking down and drying of mushrooms, and assuming features different from typhoid and marsh-malarial fevers, the conclusion seems just that there is something more than coincidence in these things. The opinion of cause and effect is greatly strengthened by some co-existing circumstances in 1847 as well as 1879.

The Salisbury theory of marsh malarial fever, which has been ridiculed almost into oblivion, perhaps unjustly, was founded on its cryptogamous origin. If true, the fungus is, of course, belonging to a different variety than that found on the hills producing the two-weeks fever, the name of which heads this article; or undergoes modification by being drifted by washings of fields and current of streams to the marsh, from which its spores are wafted through the atmosphere. This difference or modification is seen in the difference of character in the disease produced by them. While marsh malarial fever is intermittent or remittent, and cut short by quinine, the highland malarial or mushroom fever runs its usual course despite large quantities of quinine, and does not usually observe diurnal intermissions nor remissions. No abortive treatment has yet been discovered for the highland two-weeks fever.. The course found most satisfactory is that of mercurials to a limited extent, blisters to nape of the neck, veratrum, and unloading the bowels by enemata.

The various varieties of "the fever" certainly are the result of a poisoned or in some way deranged condition of the nervous centers at some point, as poisons as well as mild remedies affect some particular portion of the body by an elective tendency to that organ. Not only is their action confined to a particular organ, but even to a particular part of a system. Thus strychnine affects the spinal center of the nervous system, while opium, hyoscyamus and chloroform affect the cranial center. Now, it may be that the different poisons or their modification, affect particular portions of the nervous system or even a different part of the same center. While marsh malaria deranges the spinal cord, the cause of typhoid fever evidently impresses some of the cranial centers. One being intermittent of one week's duration, and cured by spinal tonics, the other continued and runs a period of three weeks. May not the highland cryptogams affect an intermediate point, and hence intermediate in duration, etc?

